# Sulforaphane Attenuates Chronic Intermittent Hypoxia-Induced Brain Damage in Mice *via* Augmenting Nrf2 Nuclear Translocation and Autophagy

**DOI:** 10.3389/fncel.2022.827527

**Published:** 2022-03-24

**Authors:** Xiucui Li, Huiya Ying, Zilong Zhang, Zijing Yang, Cancan You, Xiaohong Cai, Zhongdong Lin, Yanfeng Xiao

**Affiliations:** ^1^Department of Pediatrics, The Second Affiliated Hospital of Xi’an Jiaotong University, Xi’an, China; ^2^Department of Pediatrics, The Second Affiliated Hospital and Yuying Children’s Hospital of Wenzhou Medical University, Wenzhou, China; ^3^Clinical Medicine, Wenzhou Medical University, Wenzhou, China

**Keywords:** chronic intermittent hypoxia, neurocognition dysfunction, sulforaphane, nuclear factor erythroid-related factor 2, autophagy, oxidative stress, apoptosis

## Abstract

Obstructive sleep apnea–hypopnea syndrome (OSAHS), typically characterized by chronic intermittent hypoxia (CIH), is associated with neurocognitive dysfunction in children. Sulforaphane (SFN), an activator of nuclear factor E2-related factor 2 (Nrf2), has been demonstrated to protect against oxidative stress in various diseases. However, the effect of SFN on OSAHS remains elusive. In this research, we investigated the neuroprotective role of SFN in CIH-induced cognitive dysfunction and underlying mechanisms of regulation of Nrf2 signaling pathway and autophagy. CIH exposures for 4 weeks in mice, modeling OSAHS, contributed to neurocognitive dysfunction, manifested as increased working memory errors (WMEs), reference memory errors (RMEs) and total memory errors (TEs) in the 8-arm radial maze test. The mice were intraperitoneally injected with SFN (0.5 mg/kg) 30 min before CIH exposure everyday. SFN treatment ameliorated neurocognitive dysfunction in CIH mice, which demonstrates less RME, WME, and TE. Also, SFN effectively alleviated apoptosis of hippocampal neurons following CIH by decreased TUNEL-positive cells, downregulated cleaved PARP, cleaved caspase 3, and upregulated Bcl-2. SFN protects hippocampal tissue from CIH-induced oxidative stress as evidenced by elevated superoxide dismutase (SOD) activities and reduced malondialdehyde (MDA). In addition, we found that SFN enhanced Nrf2 nuclear translocation to hold an antioxidative function on CIH-induced neuronal apoptosis in hippocampus. Meanwhile, SFN promoted autophagy activation, as shown by increased Beclin1, ATG5, and LC3II/LC3I. Overall, our findings indicated that SFN reduced the apoptosis of hippocampal neurons through antioxidant effect of Nrf2 and autophagy in CIH-induced brain damage, which highlights the potential of SFN as a novel therapy for OSAHS-related neurocognitive dysfunction.

## Introduction

Obstructive sleep apnea–hypopnea syndrome (OSAHS) is characterized by the repeated obstruction of the upper respiratory tract during sleep, which results in sleep apnea and hypopnea (Gulotta et al., 2019). It can happen in any population, especially in preschool children, and its prevalence rate ranges from 1.2 to 5.7% of the general population (Zhang et al., 2020). OSAHS has emerged as an independent risk factor that contributes to many pathological consequences, such as hypertension, metabolic disorder, and gastroesophageal reflux (Torres et al., 2015; Gaines et al., 2018; Lim et al., 2018), but one of the most severe complications of OSAHS in children is neurocognitive dysfunction. Our previous study demonstrated that cognitive dysfunction is more common in OSAHS children, and chronic intermittent hypoxia (CIH) plays a critical role in it (Cai et al., 2013). The precise mechanism of OSAHS-induced neurocognitive impairment remains ambiguous. Many pathophysiological changes have been involved in the pathogenesis of OSAHS, which include oxidative stress, autophagy, cell apoptosis, and inflammation (Cai et al., 2010; Oyarce and Iturriaga, 2018; Zhou et al., 2018).

Oxidative stress contributes to cognitive dysfunction in OSAHS children *via* repeated hypoxia–reoxygenation episodes (Duan et al., 2016). Our research team successfully simulated main pathophysiological processes of OSAHS by establishing a CIH rat model and found that 8-iso-PGF2α level in serum and brain tissue was significantly increased, which also indicated that oxidative stress was involved in the process of cognitive impairment caused by CIH (Cai et al., 2010). Nuclear factor erythroid-related factor 2 (Nrf2), a critical transcription factor, is related to regulate oxidative stress and ameliorate brain damage (Dinkova-Kostova et al., 2018; Xu et al., 2020). Under unstressed states, Nrf2 interacts with Kelch-like ECH-associated protein-1 (Keap1) and remains in the cytoplasm (Wang et al., 2017). Under oxidative stress, brain is extremely susceptible to ROS insults due to its relatively weak antioxidant mechanisms (Tarafdar and Pula, 2018). Meanwhile, Nrf2 is isolated from Keap1 and translocates into the nuclei, which activates the antioxidant response element (ARE) and alleviates the damage of neurons (Wang et al., 2017; Russo et al., 2018; Tian et al., 2019).

Under the condition of oxidative stress, autophagy reduces oxidative damage by phagocytosis or degradation of oxidative stress products (Filomeni et al., 2015; Jiang et al., 2015). Autophagy has been demonstrated to be a critical physiological process involved in regulating oxidative stress and ameliorating brain injury (Tang K. K. et al., 2019). Autophagy plays a two-sided role in nervous system diseases. Existing evidences demonstrated that in the early stage of Alzheimer’s disease (AD), autophagy held a protective function on neurons by removing abnormal intracellular proteins to maintain the balance of intracellular environment, whereas autophagy overactivation damaged neurons at the later stage of AD (Li et al., 2017). In addition, autophagy indicator LC3-II was downregulated in cadmium-induced brain injury of mice, followed alleviated cognitive function, which suggests that inhibition of autophagy protected neurons (Tang K. K. et al., 2019). Therefore, autophagy holds a critical function on nervous system injury. At present, the effect and mechanism of autophagy on neuron cells following CIH is still controversial and needs to be further studied.

Sulforaphane (SFN), naturally occurring compound extracted from cruciferous vegetables of the genus Brassica, is likely to be diffused through intestinal cells and readily absorbed due to its molecular size and lipophilicity. In addition, it was reported SFN crossed blood–brain barrier and was detected in brain tissue (Jazwa et al., 2011). SFN is associated with decreased risk of cardiovascular diseases, metabolic syndrome, and neurodegenerative pathologies (Russo et al., 2018). However, no data regarding the effectiveness of SFN on CIH-induced cognition dysfunction in mice have been reported.

The previous researches have indicated that SFN exerts neuroprotective effects through activating autophagy or transcription factor Nrf2 (Jo et al., 2014; Uddin et al., 2020). However, the effect of SFN on CIH-induced brain injury remains poorly clarified. In this research, we aimed to investigate the neuroprotective role of SFN in CIH-induced cognitive dysfunction and potential mechanisms underlying regulation of Nrf2 signaling pathway and autophagy.

## Materials and Methods

### Animals and Experimental Groups

All experimental protocols were approved by Animal Research Committee of Wenzhou Medical University (wydw 2019–068). A number of forty healthy male C57BL/6 mice (weight 18–22 g; 4 weeks) were obtained from the Experimental Animal Center (license no. SCXK 2015–0001) of Wenzhou Medical University, Zhejiang Province, China. The mice were equally and randomly divided into 4 groups (*n* = 10 per group): intermittent air group (C), CIH group, CIH with SFN treatment group (CIH + SFN), and C with SFN treatment group (SFN). The mice in C and CIH group were not subjected to SFN injection. Mice in CIH + SFN and SFN groups were intraperitoneally injected with SFN (0.5 mg/kg, Sigma-Aldrich) 30 min before CIH exposure everyday (Kang et al., 2020). All animals used in the experiment were cared for in accordance with the ethical guidelines on animal experimentation of Laboratory Animals of China National Institutes of Health.

### Chronic Intermittent Hypoxia Exposures

Chronic intermittent hypoxia model simulating medium-severe OSAHS was performed as previously described with the modifications (Cai et al., 2014). To establish hypoxia–reoxygenation condition, a chamber with nitrogen–oxygen delivery system was applied following the preset protocol under the control of computer. In brief, O_2_ concentration fluctuated from 21.0 ± 0.5 to 9 ± 1.5% in intermittent hypoxia chamber. The cycle was conducted every 90 s over 7 h (from 8:00 to 15:00 in light) per day for 4 weeks. Mice in CIH and CIH + SFN groups were placed in CIH chamber, whereas those in C and SFN groups were placed in a cabin filled with compressed air, where O_2_ concentration was kept at 21.0 ± 0.5%.

### 8-Arm Radial Maze Test

To evaluate spatial memory, the 8-arm radial maze training and test was conducted after CIH as previously described (Zhang et al., 2020). During the experiment, indoor temperature was adjusted to 24 ± 2°C and humidity was maintained at 40–70% with the 12-h light–dark cycle. Mice in each group were restricted from eating to maintain 80–85 percent of their original body weight and were given free access to water throughout the trial. Food pellets were positioned at the end of 2, 4, 6, and 8 arms and were maintained in the same place during the testing phase and habituation process. At the beginning of the test, mice covered by an opaque box were placed in the center of the maze for 15 s, and then, mice were released for free feeding when the box was removed. The hind paws of mice completely enter into one arm is considered that arm entry was completed. A trial was terminated immediately when all pellets were consumed or 5 min had elapsed. After finishing the training once per day for 10 days, 8-arm radial maze test was performed as described above following CIH on day 28. The performance of mice was evaluated according to the number of error choices as follows: working memory errors (WME: re-entry into arms where food pellet had been eaten), reference memory errors (RME: entry into a never-baited arm), and the total errors (TE: mice enter a baited arm and eat the pellet as the right choice, otherwise it was counted as an error).

### Brain Tissue Collection and Preparation

Brain tissues were obtained after CIH. Totally, five mice randomly selected from each group were anesthetized with 10% chloral hydrate *via* intraperitoneal injection. Their brain tissues were dissected into left and right hemispheres immediately on ice, and left hippocampus tissues were isolated and frozen at −80°C for western blotting analysis, whereas right hippocampus was used to detect superoxide dismutase (SOD) and malondialdehyde (MDA). Subsequently, the remaining five mice in each group were dissected into left and right hemispheres by the same way. The left brains were placed into 4% paraformaldehyde for TUNEL assay and immunofluorescence, whereas the right hippocampus of the rest mice was dissected and cut into 1 × 1 × 1 cm and fixed in 4% glutaraldehyde for 24 h, followed by the observation of autophagosomes by electron microscopy.

### Transmission Electron Microscopy

Transmission electron microscopy was performed for autophagosomes observation. Hippocampus of mice was fixed with 2.5% glutaraldehyde and 2.0% paraformaldehyde in 0.1 M sodium cacodylate buffer, followed by the incubation in 1% osmium tetroxide-0.1 M sodium cacodylate buffer and dehydration by gradient alcohol and acetone. Then, the tissues were embedded before ultrathin sections were applied to cut them. After staining with uranyl acetate and lead citrate, the sections were examined under electron microscopy to assess the autophagosomes.

### Terminal Transferase-Mediated dUTP Nick End Labeling (TUNEL) Evaluation

Apoptosis detection was conducted *via* TUNEL assay kit, following the instructions. Briefly, the paraffin-embedded tissues used for TUNEL assay were sliced into 4-μm-thick sections and then dewaxed in xylene. After infiltration in 3% hydrogen peroxide for 15 min, sections were placed in TUNEL reaction mixture for 0.5 h and treated with converter-POD solution for 1 h. Sequentially, 3,3-diaminobenzidine tetra-hydrochloride (DAB) and hematoxylin dye were applied to stain and visualize the slides. TUNEL staining was performed on five consecutive sections for one specimen. After processing and dehydration, the slides were mounted with Eukitt (Sigma-Aldrich) and observed under a light microscope (OLYMPUS 1 × 70-SIF2, Japan). Cells with brownish-yellow particles in nuclei were considered as apoptotic cells. The total number of TUNEL-positive staining cells in the hippocampal CA1 region was calculated in three randomly selected views from each section and five sections per sample under a light microscope with 400 × magnification. Apoptosis index was acquired *via* the following formula: apoptosis index (AI) = the number of apoptotic cells/the number of total cells × 100%.

### Immunofluorescence

After fixation with 4% paraformaldehyde (PFA) for 12–24 h, the brain tissues were embedded in paraffin and sectioned, followed by dewaxing and rehydration. Then, sections were incubated with 0.3% Triton X-100, blocked in 10% goat serum at room temperature for 1 h, and incubated at 4°C overnight with primary antibodies: rabbit anti-Nrf2 (1:500, Affinity), rabbit anti-p62 (1:800, Cell Signaling), mouse anti-Keap1 (1:200, Abcam), rabbit anti-LC3B (1:200, Cell Signaling). Next day, fluorescence-conjugated secondary antibodies that include Alexa Fluor 488 donkey anti-rabbit IgG (1:400) or Alexa Fluor 594 donkey anti-mouse IgG (1:400) were applied to incubate the samples for an hour. The slides were sequentially mounted with DAPI and visualized through fluorescence microscopy.

### Malondialdehyde and Superoxide Dismutase Assay

The freshly collected hippocampus tissues were homogenized to measure SOD activity and MDA level. The detailed testing methods of SOD and MDA were carried out according to the manufacturer’s protocols (Beyotime Biotechnology, China).

### Cytoplasmic and Nuclear Protein Extraction

Cytoplasmic and nuclear proteins were prepared *via* Nuclear and Cytoplasmic Protein Extraction Kit (Beyotime Biotechnology, China), following the instructions. First, the hippocampus tissues were homogenized, which were cut into small pieces with cytoplasmic protein extraction reagents A and B containing 1 mM phenylmethanesulfonyl fluoride (PMSF) (Beyotime Biotechnology, China) and incubated in an ice bath for 15 min. After centrifugation for 5 min at 1,500 rpm, the supernatant was collected as cytoplasmic protein. Following the addition of PMSF-supplemented nuclear protein extraction reagent, the precipitate was vortexed for 15–30 s at maximum speed every 2 min for a total of 30 min. Finally, the solution was centrifuged at 12,000 rpm for 10 min at 4°C, and the nuclear proteins were collected from the supernatant.

### Western Blot Assays

Hippocampal tissues from each group were homogenized in ice-cold radio immunoprecipitation assay (RIPA) lysis buffer (50 mM Tris pH 7.4, 150 mM NaCl, 1% Triton X-100, 1% sodium deoxycholate, and 0.1% SDS) containing 1 mM phenylmethanesulfonyl fluoride (PMSF) (Beyotime Biotechnology, China). After the collection of supernatant, protein concentrations were measured *via* BCA Protein Assay Reagent Kit (Beyotime Biotechnology, China). Equal amounts of protein (40 μg) were electrophoresed and separated through sodium dodecyl sulfate–polyacrylamide gels (SDS-PAGE) and then transferred to PVDF membrane. After being blocked with 5% skim milk for 2 h at room temperature, the membrane was incubated overnight at 4°C with the following primary antibodies: rabbit anti-Nrf2 (1:1,000, Affinity), mouse anti-Keap1 (1:1,000, Abcam), rabbit anti-SQSTM1/p62 (1:1,000, Cell Signaling), rabbit anti-LC3B (1:1,000, Cell Signaling), rabbit anti-ATG5 (1:1,000, Abcam), rabbit anti-Beclin1 (1:2,000, Abcam), rabbit anti-cleaved -caspase 3 (1:1,000, Cell Signaling), rabbit anti-Bcl-2 (1:1,000, Affinity), rabbit anti-PARP (1:1,000, Cell Signaling), rabbit anti-HO-1 (1:1,000, Affinity), rabbit anti-NQO1 (1:1,000, Affinity), and mouse anti-β-actin (1:5,000, Cell Signaling). Next day, membranes were incubated for 1 h at room temperature with horseradish peroxidase-conjugated secondary antibodies, followed by washing with Tris-buffered saline and Tween 20 (TBST). To visualize the protein bands and analyze their intensity, an enhanced chemiluminescence detection system and ImageJ software (National Institutes of Health, Bethesda, MD, United States) were applied.

### Statistical Analyses

All data are normally distributed and expressed as means ± SD. Comparisons between groups with different treatments were analyzed *via* analysis of variance (ANOVA), followed by LSD or Dunnett’s T3 *post hoc* analysis. All statistical analyses were conducted by SPSS software (IBM Corp., Armonk, NY, United States). The statistical significance level for all comparisons was set at 0.05.

## Results

### Sulforaphane Alleviates Chronic Intermittent Hypoxia-Induced Cognitive Dysfunction in the 8-Arm Radial Maze Test

**FIGURE 1:**
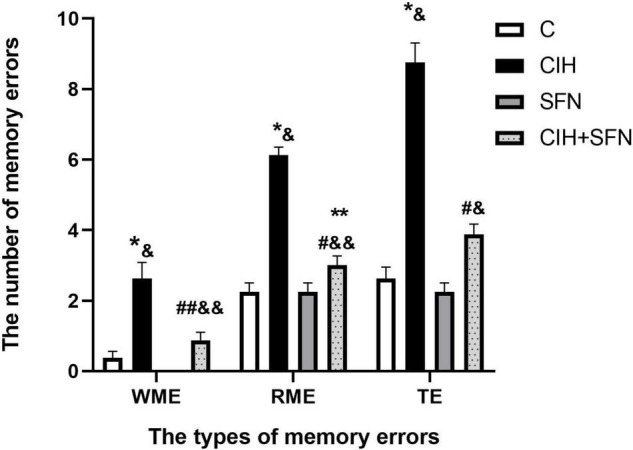
SFN alleviates CIH-induced cognitive dysfunction in the 8-arm radial maze test. The numbers of memory errors of WME, RME, and TE in different groups in 8-arm radial maze test. Graphs display normalized results of mean ± SD for *n* = 10 and statistical significance (*p ≤ 0.01 vs. C; **p ≤ 0.05 vs. C; ^#^*p* ≤ 0.01 vs. CIH; ^##^*p* ≤ 0.05 vs. CIH; ^&^*p* ≤ 0.01 vs. SFN; ^&⁣&^*p* ≤ 0.05 vs. SFN). C, intermittent air; CIH, chronic intermittent hypoxia; SFN, C with SFN treatment; CIH + SFN, CIH with SFN treatment; WME, working memory errors; RME, reference memory errors; TE, total errors.

The 8-arm radial maze test is a widely recognized method to detect learning and memory. In this study, we used 8-arm maze to evaluate the effect of CIH on cognitive function. The number of WME, RME, and TE increased significantly after intermittent hypoxia exposure (WME: *p* = 0.007, RME: *p* < 0.001, TE: *p* < 0.001, CIH vs. C group), and the above error decreased significantly after SFN pretreatment (*p* = 0.035, *p* < 0.001, *p* < 0.001, CIH + SFN vs. CIH group). Compared with SFN group, the number of memory errors significantly increased in CIH + SFN group (WME: *p* = 0.031, RME: *p* = 0.042, TE: *p* = 0.005). When compared to C group, the number of WME and TE in CIH + SFN group was no statistical difference (*p* = 0.458, 0.069), except for the increased RME (*p* = 0.042, CIH + SFN vs. C group). There was no significant difference between the SFN group and the control group. Therefore, it was found that SFN can reduce significantly the cognitive impairment in the intermittent hypoxia mice (Figure 1).

### Sulforaphane Reduces Caspase 3-Dependent Neuronal Apoptosis in Hippocampal CA1 Region

**FIGURE 2:**
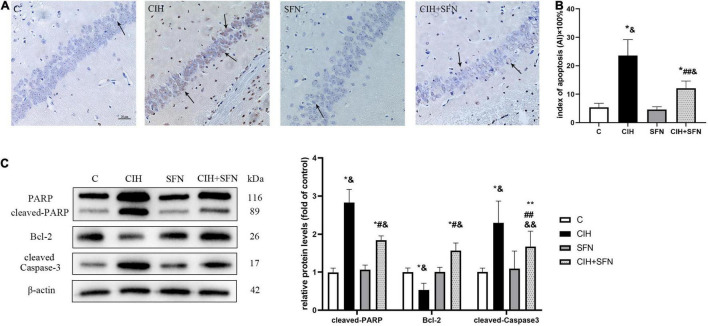
SFN reduces caspase 3-dependent neuronal apoptosis in hippocampal CA1 region. **(A,B)** Neuronal apoptosis in hippocampal CA1 region. Apoptotic neurons were detected using TUNEL staining. Arrows indicate apoptotic neurons. Magnification ×400. Bar: 20 μm. AI: apoptotic index. **(C)** Representative immunoblots and analysis of cleaved PARP, cleaved caspase 3 and Bcl-2 in each group from the hippocampus. *n* = 5, *p ≤ 0.01 vs. C; **p ≤ 0.05 vs. C; ^#^*p* ≤ 0.01 vs. CIH; ^##^*p* ≤ 0.05 vs. CIH; ^&^*p* ≤ 0.01 vs. SFN; ^&⁣&^*p* ≤ 0.05 vs. SFN.

Neural apoptosis is one of the important mechanisms associated with CIH hippocampal injury. In our study, compared with control mice, CIH resulted in an increase of TUNEL-positive neurons in the hippocampal CA1 region with nuclear concentration, whereas SFN treatment significantly reduced the apoptosis of neurons following CIH. In addition, the calculated apoptosis index by TUNEL staining in hippocampal CA1 neurons was significantly reduced in CIH + SFN group compared with CIH group (*p* = 0.032). Compared with control or SFN group, the apoptosis index in CIH + SFN was increased (*p* = 0.01, CIH + SFN group vs. SFN group; *p* = 0.007, CIH + SFN group vs. C group). There was no significant difference between groups C and SFN (Figures 2A,B).

Cleavage of poly (ADP-ribose) polymerase (PARP) is an effective criterion for determining apoptosis in cells (Li et al., 2021). We detected the level of cleaved PARP to assess apoptosis. Cleaved caspase 3, the active form of caspase 3, is recognized as an indicator of apoptosis because it is elevated during cell death (Adams and Cory, 1998). The expression level of cleaved caspase 3 reflected the severity of apoptosis. Bcl-2 exists in mitochondria as homodimer or heterodimer, which can prevent cell apoptosis. We detected cleaved PARP, cleaved caspase 3 and Bcl-2 expression after 4 weeks of CIH to investigate the possible association between SFN and hippocampal neuron apoptosis. As shown in Figure 2C, compared with C group, the level of cleaved PARP and cleaved caspase 3 was upregulated, and Bcl-2 was downregulated in CIH group (all *p* < 0.001). Compared with CIH group, SFN pretreatment during hypoxia inhibited cleaved PARP level (*p* < 0.001), cleaved caspase 3 level (*p* = 0.033), and elevated Bcl-2 expression (*p* < 0.001). These data suggested that SFN could significantly reduce CIH-induced caspase 3-dependent cell apoptosis.

### Sulforaphane Resists Chronic Intermittent Hypoxia-Induced Oxidative Stress in Hippocampus of Mice

**FIGURE 3:**
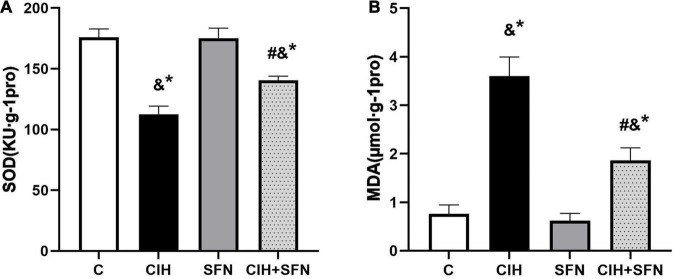
SFN resists CIH-induced oxidative stress in hippocampus of mice. Effects of SFN treatment on hippocampal tissue lysates SOD **(A)** and MDA **(B)** levels in the mice subjected to CIH exposure. Data were represented as the means ± SD, *n* = 5. *p < 0.01 vs. C; ^#^*p* < 0.01 vs. CIH; ^&^*p* ≤ 0.01 vs. SFN.

To verify that CIH can lead to oxidative stress, antioxidant enzyme SOD and lipid peroxidation product MDA level were detected in our study. As shown in Figure 3, compared with C group, SOD level was decreased (*p* < 0.001) and MDA level was increased (*p* < 0.001) in hippocampus of CIH group, whereas the results were contrary after SFN pretreatment (all *p* < 0.001, CIH + SFN vs. CIH group). SFN plays a protective role by significantly reducing the level of MDA and increasing SOD, which suggests that SFN can reduce oxidative stress in hippocampus.

### Sulforaphane Activates Nrf2-Antioxidant Response Element by Enhancing Nrf2 Nuclear Translocation After Chronic Intermittent Hypoxia

**FIGURE 4:**
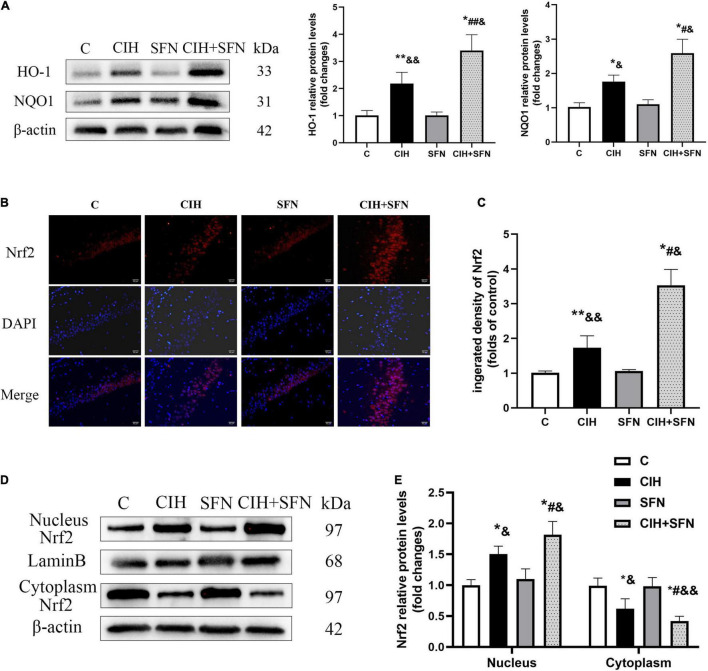
SFN activates Nrf2-ARE by enhancing Nrf2 nuclear translocation after CIH. **(A)** Representative immunoblots and analysis of HO-1 and NQO1 in each group from the hippocampus. **(B,C)** Representative immunofluorescence images demonstrating nuclear translocation of Nrf2 in hippocampal CA1 region after CIH exposure with or without SFN pretreatment. Scale bar, 20 μm. **(D,E)** Representative immunoblots and analysis of nucleus and cytoplasm Nrf2 in each group from the hippocampus. *n* = 5, *p ≤ 0.01 vs. C; **p ≤ 0.05 vs. C; ^#^*p* ≤ 0.01 vs. CIH; ^##^*p* ≤ 0.05 vs. CIH; ^&^*p* ≤ 0.01 vs. SFN; ^&⁣&^*p* ≤ 0.05 vs. SFN.

Nrf2-ARE signaling cascade is the key to regulate antioxidant genes transcription. To investigate the effect of SFN on Nrf2 pathway following CIH, first we detected antioxidative proteins (HO-1 and NQO1) of Nrf2 downstream by western blotting. Compared with C group, HO-1 protein levels in CIH group were slightly increased (*p* = 0.014), whereas treatment with SFN significantly elevated HO-1 levels following CIH (*p* = 0.034; Figure 4A). Similar results were obtained when we measured NQO1 protein levels (Figure 4B).

In addition, to further investigate the mechanisms of SFN on regulating Nrf2 signaling pathway, we evaluated Nrf2 location by immunofluorescence staining (Figure 4B). In C group, Nrf2 showed a predominant distribution in the cytoplasm, whereas in CIH group, Nrf2 was slightly accumulated in nucleus, which suggests that Nrf2 nuclear translocation occurred after CIH exposure in the hippocampus. Meanwhile, in CIH + SFN group, the intranuclear accumulation of Nrf2 was obviously increased compared with CIH group. We calculated the fluorescence density of Nrf2. As shown in Figure 4C, the fluorescence density of Nrf2 was significantly increased in CIH + SFN group than that in IH group (*p* < 0.001). These results suggested that SFN leads to the increase of nuclear Nrf2 in hippocampus under intermittent hypoxia exposure. Moreover, we evaluated the nuclear translocation of Nrf2 by extracting and analyzing cytoplasmic and nuclear proteins. As shown in Figures 4D,E, nuclear Nrf2 levels increased and cytoplasmic Nrf2 decreased after CIH compared with group C (all *p* < 0.001), and this effect was more obvious after SFN administration (*p* = 0.006, *p* = 0.027). These data revealed that SFN alleviates oxidative stress by enhancing Nrf2 nuclear translocation and its downstream ARE products following CIH.

### Sulforaphane Modulates Autophagy Induced by Chronic Intermittent Hypoxia

**FIGURE 5:**
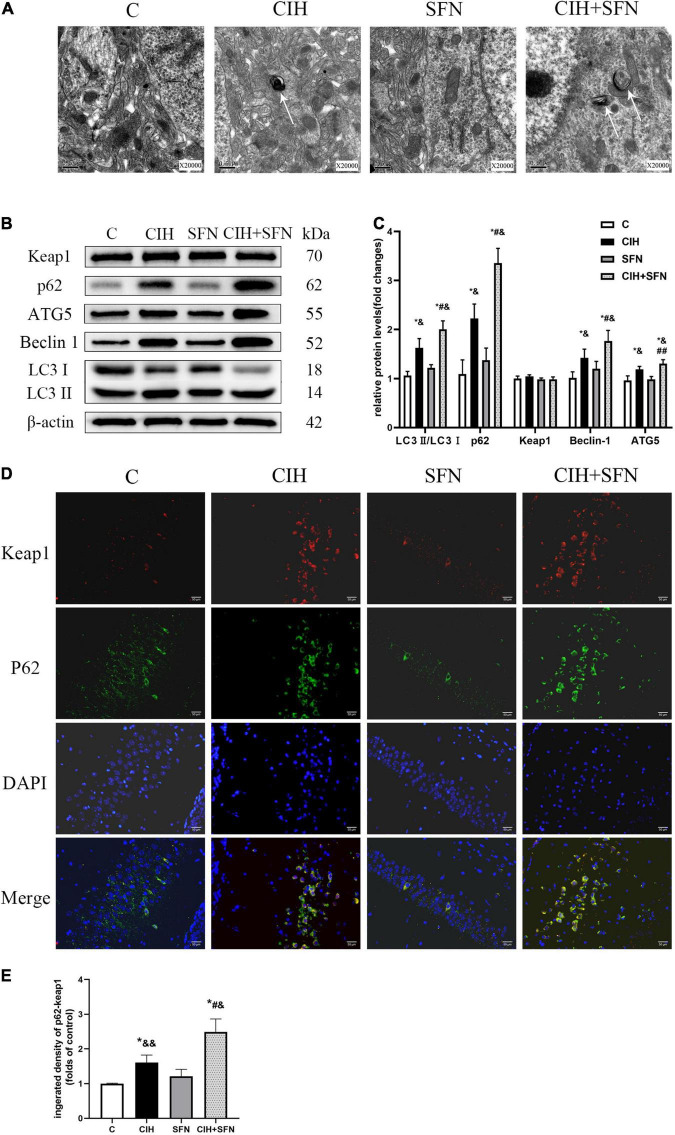
SFN modulates autophagy induced by CIH. **(A)** Transmission electron microscope reveals autophagosomes (white arrows) in hippocampal neurons. Scale bar, 0.5 μm. Magnification × 20,000. **(B,C)** Representative immunoblots and analysis of Beclin1, ATG5, LC3 II, LC3 I, p62, and Keap1 in each group from the hippocampus. **(D,E)** Representative images of p62 (green) and Keap1 (red) colocalization in hippocampal CA1 region with or without SFN treatment. Scale bar, 50 μm. *n* = 5, *p ≤ 0.01 vs. C; **p ≤ 0.05 vs. C; ^#^*p* ≤ 0.01 vs. CIH; ^##^*p* ≤ 0.05 vs. CIH; ^&^*p* ≤ 0.01 vs. SFN; ^&⁣&^*p* ≤ 0.05 vs. SFN.

To explore whether the protective effect of SFN on CIH-induced apoptosis was related to autophagy, transmission electron microscopy was used in this study to observe that the autophagosome increased slightly in CIH group, whereas the autophagosome increased significantly after SFN treatment (Figure 5A). The levels of several markers relevant to autophagy (Beclin1, ATG5, and LC3B) were determined in this research by western blotting. As shown in Figures 5B,C, CIH increased Beclin1, ATG5, LC3 II/I, and p62 protein levels compared with the control group (all *p* < 0.001), which suggests autophagy flux inhibition in hippocampus. Meanwhile, the levels of LC3 II/I and p62 were significantly increased in CIH + SFN group, compared with CIH group (all *p* < 0.001). These results suggested that SFN could increase CIH-induced autophagy. In addition, we also found that total Keap1 levels did not change significantly after SFN treatment (*p* = 0.058; Figure 5C), but immunofluorescence staining demonstrated that SFN treatment increased p62-Keap1 colocalization following CIH (*p* < 0.001, CIH + SFN vs. CIH group; Figures 5D,E).

## Discussion

Our study is the first to investigate whether SFN could protect against neurocognitive dysfunction in CIH-induced brain damage of mice. Herein, we provided the evidence that SFN exerted oxidation resistance by promoting Nrf2 nuclear translocation and regulated autophagy to alleviate CIH-induced neuronal apoptosis in hippocampus. Therefore, these data highlighted the therapeutic potential of SFN in neurocognitive dysfunction caused by CIH exposure.

Neurocognitive function is an advanced function of the brain. As we all known, prefrontal cortex and hippocampal CA1 region are cardinal brain regions associated with spatial memory (Mehta, 2015). Numerous studies have revealed that CIH is an independent risk factor for cognitive dysfunction *via* promoting neuronal apoptosis in hippocampal CA1 region (Manczak et al., 2018; Li et al., 2020). Our study showed that spatial memory errors (RME, WME, and TE) were significantly increased after CIH exposure *via* an 8-arm maze test, whereas the neuronal apoptosis of hippocampal CA1 region was consistent with the spatial memory errors in the mice, which indicates CIH-induced memory impairment by apoptosis of hippocampal CA1 region.

Chronic intermittent hypoxia-induced brain damage has been demonstrated to associate with endoplasmic reticulum stress, oxidative stress, or neuron apoptosis (Horne et al., 2018; Oyarce and Iturriaga, 2018). Particularly in CIH situations, oxidative stress plays a critical role in neurocognitive dysfunction (Tarafdar and Pula, 2018). In the recent years, autophagy has been demonstrated to reduce oxidative damage in a variety of disease models and hold a significant function on CIH-induced brain damage (Filomeni et al., 2015; Tang K. K. et al., 2019). In addition, Nrf2 signaling pathway represents a significant mechanism that underlies preventing against oxidative stress and allowing for further adjustments in CIH-induced brain damage (Zolotoff et al., 2020). However, the treatment of CIH-induced neurocognitive dysfunction in clinical application is still very lacking, and many agents include questionable side effects. Therefore, further development of safe and efficacious drugs for the treatment of CIH-induced brain injury is needed.

Sulforaphane is an isothiocyanate derived from glucoraphanin. In neurological diseases, the prophylactic and therapeutic effects of SFN, which is associated with reduced risk of learning and memory impairment, have been revealed (Uddin et al., 2020). It has been reported that SFN can retard the progress of memory impairment in nervous system diseases such as AD and cerebral ischemia, *via* inhibition of oxidative stress, apoptosis, and neuroinflammation (Huang et al., 2019). In this study, we paid attention to apoptosis in hippocampal CA1 region and found SFN inhibited the caspase 3-dependent apoptosis of hippocampal neurons induced by CIH to hold a neuroprotective function. It is reported that oxidative stress is involved in inducing apoptosis in nervous system diseases (Coimbra-Costa et al., 2017). we all know that the brain contains a lot of unsaturated fatty acids, which are vulnerable to oxidative stress. Therefore, MDA, a biomarker of lipid peroxidation, was detected to demonstrate CIH-induced oxidative stress. In addition, our previous study and other researches have also proved that lipid peroxidation is related to memory damage caused by CIH (Gozal et al., 2010; Li et al., 2018). To determine the effect of SFN on oxidative stress hippocampal neurons, we exposed SFN pretreated mice to CIH and found that SFN elevated activities of SOD and reduced MDA in hippocampal neurons. In summary, these data provided strong evidence that SFN improved CIH-induced neurocognitive dysfunction *via* attenuating oxidative stress and apoptosis.

Sulforaphane is known as natural agonist to activate Nrf2 that regulates antioxidant enzymes to protect cells from oxidative stress and is critical to alleviate the damage of neurons. In this research, we demonstrated that SFN activated Nrf2 pathway and promoted Nrf2 nuclear translocation after CIH to alleviate oxidative damage. In addition, we found that Nrf2 nuclear translocation and the levels of ARE (HO-1 and NQO1), downstream of Nrf2 signal pathway, were significantly increased following CIH in SFN-pretreated mice (Figures 5A,D). On the contrary, apoptosis in hippocampal CA1 region was significantly reduced. The data suggested that SFN prevented against CIH-induced neuronal apoptosis by promoting Nrf2 nuclear translocation.

P62, a multifunctional protein, has been proved to be activated Nrf2 under oxidative damage (Katsuragi et al., 2015). The recent studies have shown p62 links to Nrf2 signaling pathway and autophagy (Jiang et al., 2015). Moreover, under oxidative stress, p62 was found to bind competitively with Keap1 to increase Nrf2 nuclear translocation, then Nrf2 activated downstream target genes that include p62, and p62 was further phosphorylated to increase Nrf2 nuclear translocation, which forms a positive feedback loop; nevertheless, overexpression of Nrf2 plays a damaging role (Silva-Islas and Maldonado, 2018). In this experiment, we found that there was a large accumulation of P62 in hippocampus of CIH mice with SFN treatment, and the binding of p62-Keap1 was enhanced (Figures 5D,E), followed by increased nuclear translocation of Nrf2. The p62-keap1 complex is constantly degraded by autophagy to inhibit excessive Nrf2 expression; therefore, autophagy acts a response to prevent the damage caused by excessive Nrf2 expression and oxidative stress (Jiang et al., 2015; Tang Z. et al., 2019).

In our study, we found that SFN treatment attenuated CIH-induced apoptosis and contributed to significant p62 accumulation, autophagy, and nuclear translocation of Nrf2. Meanwhile, SFN treatment also increased p62-Keap1 colocalization following CIH. Autophagy may exert an antioxidant effort activated by a Keap1–Nrf2–p62 feedback loop (Tang Z. et al., 2019), so we hypothesized that SFN increased Nrf2 nuclear translocation by increasing autophagy degradation of Keap-1 and p62 to reduce oxidative stress, which held a protective function. However, the specific mechanism still needs further investigation. Besides, we found caspase 3-dependent apoptosis was significantly activated in CIH exposure condition, although autophagy and antioxidant products were slightly increased to resist CIH, the outcome of apoptosis cannot be reversed.

## Conclusion

In summary, the present data provide the first evidence that SFN exerted a definite protective effect on reducing apoptosis of hippocampal neurons through antioxidant effect of Nrf2 and autophagy in CIH-induced brain damage. Furthermore, the detail relationship between Nrf2 and autophagy in CIH needs further study. Our study highlights the potential of SFN as an adjuvant therapy for OSAHS, and further investigation is needed to confirm the therapeutic effect of SFN in clinic.

## Data Availability Statement

The original contributions presented in the study are included in the article/supplementary material, further inquiries can be directed to the corresponding author/s.

## Ethics Statement

The animal study was reviewed and approved by the Animal Management and Ethics Committee.

## Author Contributions

XL designed the experiment and wrote the manuscript. HY, CY, and ZY completed part of the experiment. ZZ drew the graph. XC and ZL directed the experiment design. YX directed and revised the manuscript. All authors contributed to the article and approved the submitted version.

## Conflict of Interest

The authors declare that the research was conducted in the absence of any commercial or financial relationships that could be construed as a potential conflict of interest.

## Publisher’s Note

All claims expressed in this article are solely those of the authors and do not necessarily represent those of their affiliated organizations, or those of the publisher, the editors and the reviewers. Any product that may be evaluated in this article, or claim that may be made by its manufacturer, is not guaranteed or endorsed by the publisher.
